# The British Medical Association on the Registration of Infectious Diseases

**Published:** 1879-10

**Authors:** H. M. Lyman


### Domestic Correspondence.

### ARTICLE XII.

### THE BRITISH MEDICAL ASSOCIATION ON THE REGISTRATION OF INFECTIOUS DISEASES.

No one can accuse our British medical brethren of any lack of zeal regarding the prevention of disease. Antiseptic surgery and public health are their favorite watchwords. But, though anxious enough for acts of parliament and for local legislation, to " stamp out " disease, they do not propose to forget that a physician is, *ex-officio*, a gentleman, and that he must be treated like a gentleman. In several of the large towns of the United Kingdom, the authorities have borrowed a Chicago idea, and have passed laws requiring a physician to report all infectious diseases in their practice. Disobedience to be punished by fines ranging from ten to fifty dollars; while obedience was to be rewarded with a fee of two shillings and sixpence or three shillings and sixpence for each report.

To the credit of our profession be it said, this attempt aroused the medical lions at once. The greatest indignation was expressed, and the British Medical Association took up the matter at its recent meeting in the city of Cork. The committee to which the subject was referred, after reciting the above facts, proceeded as follows (*British Medical Journal*, Aug. 16, 1879) :

" It is greatly to be deplored that the suggestion has not been followed that was made in the first instance by this committee, namely, that the householder, or person in charge of the infectious case, should be the person required to notify the case to the medical officer of health.

" The importance of an early notification of the commencement of an epidemic is so great, that the undertaking should never have been imperiled by the enactment of obnoxious and entirely unnecessary penal clauses against medical men; and in several towns, it has already roused against it the strenuous opposition of members of the profession, by whose willing aid alone it could be carried into effect.

"There are several grave objections to the proposal to make the medical attendant primarily responsible to the local authority for the notification of the presence of infectious disease in the households of their patients.

"1. Whatever may be said as to the abstract right of the state to require this service, it is most undesirable that the confidential relation of the medical attendant and patient should be disturbed, or that the former should be placed in the position of an *informer* in reference to the affairs of the latter.

"2. It is not well that, in addition to the heavy responsibilities and risk to life attaching to attendance upon cases of infectious disease, medical men should be liable to vexatious actions at law, and to heavy penalties for alleged non-performance of a duty that lies outside of their ordinary practice, and that would be more justly and better performed by persons in charge of the cases.

"3. Such an enactment would also often tend to defeat its own ends, seeing that it would in many cases prevent resort to medical assistance, or lead to the employment of unqualified practitioners, and to more frequent application to druggists. Patients suffering from these complaints, would thus not only run much greater danger to life, but the community would be more frequently exposed to danger of infection.

"4. Any injury to trade, or to the means of gaining a livelihood, in consequence of this notification, would probably be ascribed to the action of the medical attendant, and, in case of a disputed diagnosis, might lead to further evils than mere loss of practice.

"5. It is not desirable that the extent of a medical man's practice in these cases should be made known to, and commented upon by members of the various local government boards. None of those objections apply to the plan proposed by your committee; and if the returns of disease made by householders were sent direct to the medical officer of health, and were considered by him as confidential, the measures necessary for the protection of the neighbors could be carried out without unnecessary publicity, and much needless alarm on the part of the community would be avoided. It has been suggested that in poor districts, it might be very difficult to obtain from ignorant and illiterate persons the necessary announcement of the nature of the case; but the authoritative declaration of the nature of the case must necessarily come, in the first instance, from the medical attendant, and, if he were provided with a form that could be countersigned and forwarded by the householder, the responsibility of the latter for the notification of the case would remain with him; the medical man would simply have done his duty towards his patient.

"Inasmuch as this service would be rendered also to the local authority, he might reasonably claim to be remunerated by them for the certificate."

This report was warmly supported by the Association, and measures for carrying its recommendations into effect are to be initiated in Parliament. Such an exhibition of highly civilized feeling is extremely creditable to our English brothers. Occurring as it does at a long distance from our own country, perhaps our sanitary enthusiasts will condescend to take under consideration the lesson which it illustrates. Are we to be the only representatives of a learned profession, in an enlightened country, who will tamely submit to impositions against which the "down-trodden subjects of an effete monarchy" instinctively revolt?

H. M. LYMAN.

